# A Distributed Fault Diagnosis and Cooperative Fault-Tolerant Control Design Framework for Distributed Interconnected Systems

**DOI:** 10.3390/s22072480

**Published:** 2022-03-23

**Authors:** Xue Li, Zhikang Fan, Shengfeng Wang, Aibing Qiu, Jingfeng Mao

**Affiliations:** School of Electrical Engineering, Nantong University, Nantong 226019, China; xueleey@163.com (X.L.); a1024584761@163.com (Z.F.); wangsf@ntu.edu.cn (S.W.); mao.jf@ntu.edu.cn (J.M.)

**Keywords:** distributed fault diagnosis, fault isolation, cooperative fault-tolerant control, distributed interconnected systems

## Abstract

This paper investigates a design framework for a class of distributed interconnected systems, where a fault diagnosis scheme and a cooperative fault-tolerant control scheme are included. First of all, fault detection observers are designed for the interconnected subsystems, and the detection results will be spread to all subsystems in the form of a broadcast. Then, to locate the faulty subsystem accurately, fault isolation observers are further designed for the alarming subsystems in turn with the aid of an adaptive fault estimation technique. Based on this, the fault estimation information is used to compensate for the residuals, and then isolation decision logic is conducted. Moreover, the cooperative fault-tolerant control unit, where state feedback and cooperative compensation are both utilized, is introduced to ensure the stability of the whole system. Finally, the simulation of intelligent unmanned vehicle platooning is adopted to demonstrate the applicability and effectiveness of the proposed design framework.

## 1. Introduction

With the rapid development of sensing and communication technologies, modern engineering systems are increasingly networked and distributed [1]. Further, the large-scale distributed systems such as power grid and vehicle platooning are generally interconnected, physically or informationally [2,3,4,5]. These kinds of systems are thus referred to as distributed interconnected systems, which are composed of several subsystems in different locations through coupling mechanisms. On the other hand, the increasing size and complexity of distributed interconnected systems makes the occurrence of faults easier. Besides, due to the characteristics of interconnection, the fault diagnosis for distributed interconnected systems is challenging as an incipient fault occurring in any subsystem can potentially propagate from one subsystem to another and even result in the collapse of the whole system. The research on fault diagnosis and fault-tolerant control for distributed interconnected systems is receiving remarkable attention [6,7,8,9].

For the most part, the fault diagnosis approaches for distributed interconnected systems can be divided, according to the information used by the diagnostic units, into three categories: centralized, decentralized, and distributed fault diagnosis [10]. The centralized fault diagnosis approach employs a centralized diagnostic unit to collect the information of the whole system and then conducts fault diagnosis for all subsystems. In [11], an interconnected system with disconnected interconnections and packet dropouts was augmented into a switched system, and then a centralized robust fault detection filter was further designed. Obviously, the centralized approach requires high computation as well as communication and is not easy to expand, so it is not suitable for large-scale distributed interconnected systems. In the decentralized fault diagnosis approach, each subsystem is equipped with a local diagnostic unit which diagnoses its own faults with its own information; thus, the approach to interconnections among subsystems is of great importance. The conventional idea is to regard interconnections as external disturbances and design robust local observers to make local residuals insensitive to interconnections. In [12], a decentralized sensor fault isolation approach was investigated for a class of large-scale interconnected nonlinear systems. Some prior known reference signals were utilized to estimate interconnections and then the maximum impact of the estimation error on the local residual was assessed for an adaptive threshold setting. Although the decentralized approach does not need to consider information transmission or security issues, because of no interactions among subsystems, it is conservative to think of interconnections as disturbances or estimate interconnections based on prior information, which can result in a low fault detection rate.

In the distributed fault diagnosis approach, the local diagnostic unit for each subsystem can not only use information about the subsystem itself, but also interact with other subsystems. In other words, the distributed fault diagnosis approach can improve the fault diagnosis performance by partial information interaction which can obtain interconnection characteristics, and thus has received much more attention [13,14,15,16,17]. Fault isolation is the key to fault diagnosis for the distributed interconnected systems as interconnection characteristics can lead to fault propagation. In [15], fault isolation for a class of fuzzy interconnected systems was considered for the first time under the framework of interval observers, and piecewise interval observers were constructed to characterize the unknown interconnections among subsystems and the residual intervals were further used to realize fault isolation. In [16], fault isolation was achieved through the decoupling method. The unknown input consisting of faults in the other subsystems and disturbance in the whole system could be partitioned into the decoupled and the non-decoupled part, and a bank of finite-frequency $H_{-}/H_{\infty }$ unknown input observers were further constructed. Moreover, a set of linear matrix inequalities were also used to ensure that the generated residual was sensitive to the fault, while remaining robust against the unknown input. Although the method provides design with a degree of freedom, the appropriate computation capacity and resources are demanded. In addition, information interaction among subsystems can exacerbate fault propagation, especially for sensor faults. In [17], the problem of fault isolation for sensor faults was studied. The influence of local and propagated sensor faults on the residuals was analyzed to realize distributed fault isolation for multiple sensor faults in interconnected systems. However, one of the main focuses in some distributed systems is to minimize the number of measurements shared among subsystems to reduce the communication cost. Meanwhile, different strategies including the fault-driven minimal structurally overdetermined set strategy, the minimal hitting set strategy, the equation-based strategy, and a set of fault-driven minimal structurally overdetermined sets strategies have been explored [8,18,19,20]. Distinguished from what has been mentioned above, the broadcasting communication is used for information exchange in our work, and the means of communication is not the focus.

Less research effort has been made, in comparison with fault diagnosis, to study fault-tolerant control for distributed interconnected systems. The traditional fault-tolerant control approach is to estimate the faults, or the changes of the subsystems caused by the faults, approximately via the adaptive or neural networked method, and then design the distributed or decentralized local fault-tolerant control law to compensate for the faults, so that the subsystems or the whole system can recover to an acceptable performance [21,22,23]. In these schemes, fault-tolerant control only regulates the controllers of the faulty subsystems, so it is called independent fault-tolerant control. From the perspective of globally distributed interconnected systems, another fault-tolerant control approach, called cooperative fault-tolerant control, is to make full use of subsystems and the cooperative effect of their coupling mechanisms to ensure the performance of the faulty system. In [24], a novel fault-tolerant control scheme for switched and interconnected nonlinear systems was designed to guarantee the stability of the state based on “fault-tolerant control Lyapunov–Barrier functions”. In [25], the cycle-small-gain theorem was utilized to ensure the closed-loop stability of interconnected systems, and a fault-tolerant control scheme that considered both rigid and flexible component faults was proposed. However, the use of the small gain theorem generally leads to a conservative result, and the fault-tolerant objective is only to guarantee the stability of faulty systems. To the best of our knowledge, most investigations on fault-tolerant control for the distributed interconnected systems are limited to basic stability analysis, whereas other dynamic and static properties have not been covered in great detail.

Inspired by the above considerations, a distributed fault diagnosis and cooperative fault-tolerant control design framework for distributed interconnected systems is proposed in this paper. Specifically, the contributions of this paper are as follows:

1. A novel fault diagnosis framework, which is mainly composed of fault detection observers and fault isolation observers, is developed for a general class of distributed interconnected systems with actuator faults. By transmitting the state estimation information in the form of a broadcast communication and carrying out several decision logic schemes in the cloud processing unit based on the residuals to achieve fault detection, isolation, and estimation, the problem of fault propagation can be solved as well;
2. A cooperative fault-tolerant control scheme, where LQR controllers for the healthy subsystems and a cooperative fault-tolerant controller for the faulty subsystem are utilized respectively, is also proposed to guarantee the stability and performance of the whole system;
3. Different from the conventional isolation decision logic, the adaptive method is employed to estimate the fault and the fault estimation information is used to modify the residuals. In this way, the subsystem with an actuator fault can be located where the residual value is less than the threshold rather than exceeding the threshold as usual.

This paper is organized as follows. In Section 2, the framework of distributed fault diagnosis and cooperative fault-tolerant control is introduced briefly, followed by the corresponding design objective. Section 3 presents the main results, including the design of fault detection observer, fault isolation observer, and cooperative fault-tolerant controller. Section 4 is dedicated to the simulation of intelligent unmanned vehicle platooning to demonstrate the applicability and effectiveness of the proposed design scheme. Ultimately, some conclusions and possible future research directions are presented in Section 5.

## 2. Problem Description

**Figure 1:**
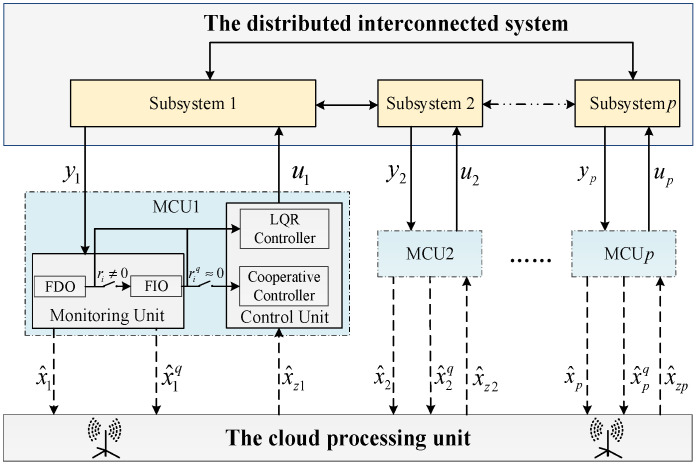
The distributed fault diagnosis and cooperative fault-tolerant control design framework.

The design framework of distributed fault diagnosis and cooperative fault-tolerant control for distributed interconnected systems is depicted in Figure 1 and mainly includes the monitoring and control units (MCUs) and cloud processing unit. The whole distributed interconnected system consists of p subsystems and is modeled as

(1) $$\left\{ \begin{array}{l} x(k+1)=Ax(k)+Bu(k)+B_{f}f(k)+B_{v}v(k)\\ y(k)=Cx(k)+Ew(k) \end{array}\right.$$

where $x(k)={\left[\begin{array}{ccccc} {x_{1}}^{T}(k) & \cdots & {x_{i}}^{T}(k) & \cdots & {x_{p}}^{T}(k) \end{array}\right]}^{T}\in {ℜ}^{n}$ denotes the state vector, with $x_{i}(k)\in {ℜ}^{n/p}$ the $i^{\mathrm{th}}$ subsystem state. $u(k)={\left[\begin{array}{ccccc} {u_{1}}^{T}(k) & \cdots & {u_{i}}^{T}(k) & \cdots & {u_{p}}^{T}(k) \end{array}\right]}^{T}\in {ℜ}^{m}$ denotes the input vector, with $u_{i}(k)\in {ℜ}^{m/p}$ the *i*th subsystem input.$y(k)={\left[\begin{array}{ccccc} {y_{1}}^{T}(k) & \cdots & {y_{i}}^{T}(k) & \cdots & {y_{p}}^{T}(k) \end{array}\right]}^{T}\in {ℜ}^{r}$ denotes the output vector, with $y_{i}(k)$ the $i^{th}$ subsystem output.$f(k)=[\begin{array}{cccc} {f_{1}}^{T}(k) & \cdots & {f_{i}}^{T}(k) & \cdots \end{array}$.${{f_{p}}^{T}(k)]}^{T}\in {ℜ}^{m}$ represents the actuator failure to be isolated, with $f_{i}(k)\in {ℜ}^{m/p}$ the *i*th actuator failure. $v(k)={\left[\begin{array}{ccccc} {v_{1}}^{T}(k) & \cdots & {v_{i}}^{T}(k) & \cdots & {v_{p}}^{T}(k) \end{array}\right]}^{T}\in {ℜ}^{m}$ stands for the process noise, with $v_{i}(k)\in {ℜ}^{m/p}$ the *i*th subsystem process noise.$w(k)={\left[\begin{array}{ccccc} {w_{1}}^{T}(k) & \cdots & {w_{i}}^{T}(k) & \cdots & {w_{p}}^{T}(k) \end{array}\right]}^{T}\in {ℜ}^{s}$ denotes the measurement noise, with $w_{i}(k)\in {ℜ}^{s/p}$ the *i*th subsystem measurement noise. A, B, $B_{f}$, $B_{v}$, C and E in Equation (1) can be decomposed into

$$A=\left[\begin{array}{cccc} A_{11} & A_{12} & \ldots & A_{1p}\\ A_{21} & A_{22} & \ldots & A_{2p}\\ \vdots & \vdots & \ddots & \vdots \\ A_{p1} & A_{p2} & \ldots & A_{pp} \end{array}\right],B=\left[\begin{array}{cccc} B_{11} & B_{12} & \ldots & B_{1p}\\ B_{21} & B_{22} & \ldots & B_{2p}\\ \vdots & \vdots & \ddots & \vdots \\ B_{p1} & B_{p2} & \ldots & B_{pp} \end{array}\right],B_{f}=\left[\begin{array}{cccc} B_{f_{1}} & 0 & \cdots & 0\\ 0 & B_{f_{2}} & \cdots & 0\\ \vdots & \vdots & \ddots & \vdots \\ 0 & 0 & \cdots & B_{f_{p}} \end{array}\right],\;B_{v}=\left[\begin{array}{cccc} B_{v_{1}} & 0 & \cdots & 0\\ 0 & B_{v_{2}} & \cdots & 0\\ \vdots & \vdots & \ddots & \vdots \\ 0 & 0 & \cdots & B_{v_{p}} \end{array}\right],C=\left[\begin{array}{cccc} C_{1} & 0 & \cdots & 0\\ 0 & C_{2} & \cdots & 0\\ \vdots & \vdots & \ddots & \vdots \\ 0 & 0 & \cdots & C_{p} \end{array}\right],E=\left[\begin{array}{cccc} E_{1} & 0 & \cdots & 0\\ 0 & E_{2} & \cdots & 0\\ \vdots & \vdots & \ddots & \vdots \\ 0 & 0 & \cdots & E_{p} \end{array}\right]$$

The *i*th subsystem can be further given as

(2) $$\left\{ \begin{array}{l} x_{i}(k+1)=A_{ii}x_{i}(k)+\sum_{j\in N_{\bar{i}}}A_{ij}x_{j}(k)+\sum_{l\in N}B_{il}u_{l}(k)+B_{f_{i}}f_{i}(k)+B_{v_{i}}v_{i}(k)\\ y_{i}(k)=C_{i}x_{i}(k)+E_{i}w_{i}(k) \end{array}\right.$$

where N is the set of all subsystems and $N_{\bar{i}}$ is the set of subsystems other than the *i*th subsystem. $x_{j}(k)$ and $u_{l}(k)$ represent the *j*th (j≠i) subsystem state and *l*th subsystem input. Note that $f_{i}(k)$ denotes the actuator failure and, in general, $B_{f_{i}}=B_{ii}$.

It can be found that each subsystem is equipped with an MCU which consists of the following components:

(1) A fault detection observer (FDO), which is governed by

(3) $$\left\{ \begin{array}{l} {\hat{x}}_{i}(k+1)=A_{ii}{\hat{x}}_{i}(k)+\sum_{j\in N_{\bar{i}}}A_{ij}{\hat{x}}_{j}(k)+\sum_{l=1}^{N}B_{il}u_{l}(k)+L_{i}(y_{i}(k)-{\hat{y}}_{i}(k))\\ {\hat{y}}_{i}(k)=C_{i}{\hat{x}}_{i}(k)\\ r_{i}(k)=y_{i}(k)-{\hat{y}}_{i}(k) \end{array}\right.$$

where ${\hat{x}}_{i}(k)$ and ${\hat{x}}_{j}(k)$ are the state estimations of the *i*th and *j*th subsystem respectively. ${\hat{x}}_{i}(k)$ represents the output estimation of the *i*th subsystem. $L_{i}$ is the detection observer gain and $r_{i}(k)$ stands for the residual of the *i*th subsystem generated by the FDO.

(2) A fault isolation observer (FIO), which is activated when there is an alarm provided by the corresponding FDO and can be described by

(4) $$\left\{ \begin{array}{l} {{\hat{x}}_{i}}^{q}(k+1)=A_{ii}{{\hat{x}}_{i}}^{q}(k)+\sum_{j\in Alarm}A_{ij}{\hat{x}}_{j}(k)+\sum_{\bar{j}\in \bar{Alarm}}A_{i\bar{j}}{\hat{x}}_{\bar{j}}(k)+\sum_{l=1}^{N}B_{il}u_{l}(k)\\ \;\;\;\;+B_{\mathrm{fi}}{\hat{f}}_{i}(k)+G_{i}(y_{i}(k)-{\hat{y}}_{i}{}^{q}(k))\\ {{\hat{y}}_{i}}^{q}(k)=C_{i}{{\hat{x}}_{i}}^{q}(k)\\ {r_{i}}^{q}(k)=y_{i}(k)-{{\hat{y}}_{i}}^{q}(k)\\ {\hat{f}}_{i}(k+1)={\hat{f}}_{i}(k)+\Gamma_{i}(y_{i}(k)-{\hat{y}}_{i}^{q}(k)) \end{array}\right.$$

where $Alarm+\bar{Alarm}=N_{\bar{i}}$ and ${{\hat{x}}_{i}}^{q}(k)$ is the state estimation of subsystem given by the FIO. $G_{i}$ is the isolation observer gain of the *i*th subsystem. ${{\hat{y}}_{i}}^{q}(k)$ represents the corresponding output estimation and ${r_{i}}^{q}(k)$ is the residual of the *i*th subsystem generated by the FIO. ${\hat{f}}_{i}(k)$ stands for the fault estimation and $\Gamma_{i}$ is the weighting matrix.

(3) A controller, which can keep the faulty system stable and is constructed as

(5) $$u_{i}(k)=\left\{ \begin{array}{l} \begin{array}{cc} -R_{i}^{-1}B_{ii}^{T}K_{i}{\hat{x}}_{zi}(k), & fault-free \end{array}\\ \begin{array}{cc} -R_{i}^{-1}B_{ii}^{T}K_{i}{\hat{x}}_{zi}(k)-R_{i}^{-1}B_{ii}^{T}S_{i}(k), & faulty \end{array} \end{array}\right.$$

where $R_{i}$ is a positive definite matrix, and $K_{i}$ is the local optimal gain determined from a standard LQR Riccati equation. ${\hat{x}}_{zi}(k)=\left\{ \begin{array}{l} \begin{array}{cc} {\hat{x}}_{i}, & fault-free \end{array}\\ \begin{array}{cc} {\hat{x}}_{i}^{q}, & faulty \end{array} \end{array}\right.$ is the real state estimation of $x_{i}(k)$ and is provided by the cloud processing unit. $S_{i}(k)$ represents the cooperative compensation vector from other subsystems in the faulty case.

**Figure 2:**
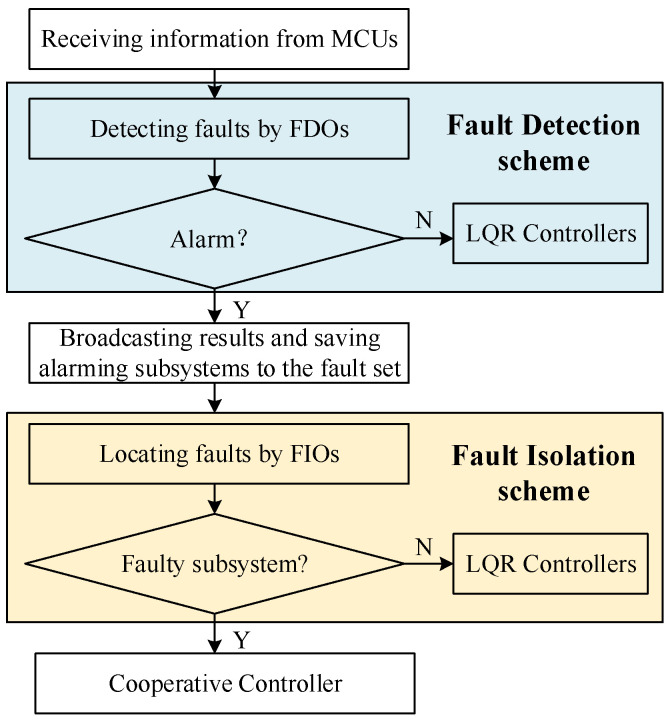
The function of the cloud processing unit.

The processing flow of the cloud processing unit is shown in detail in Figure 2. It can be seen that the clouding processing unit shoulders the responsibility of receiving, processing, and broadcasting information. Further, it mainly perform three functions: (i) obtaining the state estimations ${\hat{x}}_{i}$ and ${\hat{x}}_{i}^{q}$ from the monitoring unit; (ii) accomplishing fault detection and isolation based on the residual signals and spreading results; and (iii) providing the corresponding state estimation to the control unit.

**Figure 3:**
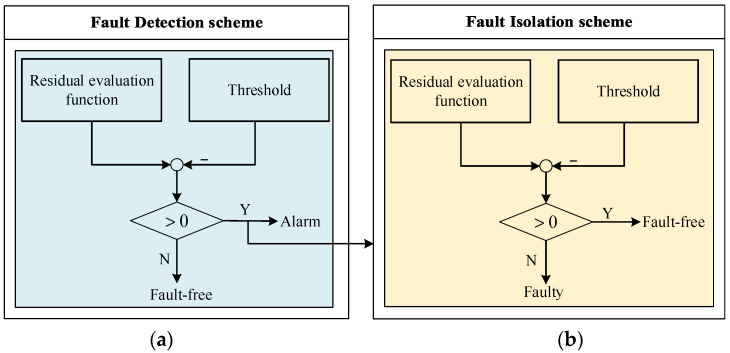
Fault detection and isolation schemes, where (**a**) represents fault detection scheme; (**b**) represents fault isolation scheme.

Based on this, the fault detection and isolation schemes in particular are given in Figure 3. The conventional fault detection observer is used to detect whether a fault occurs, and a residual value exceeding the threshold indicates that there is a fault in the process. Meanwhile, the fault isolation observer based on the adaptive fault estimation method is adopted to achieve fault isolation by the combination of an unconventional isolation decision logic. Specifically, since the adaptive method is employed to estimate the fault and the fault estimation information is used to modify the residuals. In this way, the subsystem with actuator faults can be located where the residual value is less than the threshold rather than exceed the threshold as usual. It is noteworthy that isolation decision logic in this paper is contrary to the detection decision logic and different from the conventional method [26].

In this paper, the design objective is to locate the fault accurately and achieve a cooperative fault-tolerant control. Hence, this paper studies the design of a novel fault diagnosis framework, and the detection and isolation observer gain $L_{i}$ and $G_{i}$, the controller gain $K_{i}$, and the cooperative compensation vector $S_{i}(k)$.

**Remark** **1.** *It is worthwhile to note that only the single fault case is considered in this paper. Meanwhile, a cooperative controller with fault-tolerant ability is introduced to keep the faulty subsystem stable, and LQR controllers are employed so that the healthy subsystems, which may be affected by the faulty subsystem, can remain stable.*

**Remark** **2.** *The subsystems in Figure 1 are physically interconnected. From the mathematical viewpoint, the physical interconnection can be seen from the state matrix A.* *If the matrix $A_{ij}(i\neq j)$,* *the non-diagonal block of the matrix A,* *is not equal to zero, it means that the ith* *subsystem and the jth* *subsystem are physically interconnected. In addition, the monitoring units in Figure 1 are informationally interconnected. To be specific, the monitoring units acquire the state estimation information from other interconnected subsystems through broadcast communication, and all subsystems can use them once the state estimation information has been broadcast.*

**Remark** **3.** *Fault isolation is achieved by making use of the adaptive fault estimation observer which is not applicable for a sensor fault. This is the reason we do not consider a sensor fault. If the fault isolation observer based on the adaptive fault estimation method is replaced by some other sensor fault estimation observer, the problem of sensor fault diagnosis can be considered.*

**Remark** **4.** *Similar to what has been given in [27], the necessary conditions for the existence of the observer are* $\left(A_{ii},C_{i}\right)(i=1,\cdots p)$ *being observable and* (A,C) *being observable, which can be guaranteed by the PBH rank criteria* $rank\left[\begin{array}{cc} C_{i}^{T} & sI_{i}-A_{ii}^{T} \end{array}\right]=n/p$ *and* $rank\left[\begin{array}{cc} C^{T} & sI-A^{T} \end{array}\right]=n$ *respectively.*

## 3. Main Results

The presentation of the main results is divided into three sections: (i) the design of fault detection observer; (ii) the design of fault isolation observer; and (iii) the design of cooperative fault-tolerant controller.

### 3.1. The Design of Fault Detection Observer

In the design of the fault detection observer, the only design parameter is the observer gain $L_{i}$ in Equation (3). To this end, we define the state error as $e_{i}(k){=x}_{i}(k)-{\hat{x}}_{i}(k)$ and the dynamics of the error system are obtained from Equations (2) and (3) as

(6) $$\left\{ \begin{array}{l} e_{i}(k+1)=(A_{ii}-L_{i}C_{i})e_{i}(k)+\sum_{j\in N_{\bar{i}}}A_{ij}e_{j}(k)+B_{fi}f_{i}(k)+B_{di}d_{i}(k)\\ r_{i}(k)=C_{i}e_{i}(k)+D_{i}d_{i}(k) \end{array}\right.$$

where $B_{\mathrm{di}}=\left[\begin{array}{cc} B_{vi} & -L_{i}E_{i} \end{array}\right]$, $D_{i}=\left[\begin{array}{cc} 0 & E_{i} \end{array}\right]$ and $d_{i}(k)={\left[\begin{array}{cc} {v_{i}}^{T}(k) & {w_{i}}^{T}(k) \end{array}\right]}^{T}$, and the residual $r_{i}(k)$ is related to $v_{i}(k)$ and $w_{i}(k)$. Thus, the asymptotic stability and $H_{\infty }$ performance of the error system given in Equation (6) are ensured in the following theorem.

**Theorem** **1.** *For a given scalar* $\gamma_{i}>0$ *, if there exists a symmetric matrix* $P_{i}=P_{i}^{T}>0$ *and* $Y_{i}$ *such that*

(7) $$\left[\begin{array}{ccccccccccc} -P_{i} & P_{i}A_{ii}-Y_{i}C_{i} & P_{i}B_{v_{i}} & -P_{i}L_{i}E_{i} & P_{i}A_{im} & \cdots & P_{i}A_{ip} & 0 & 0 & \cdots & 0\\ {}* & -P_{i} & 0 & 0 & 0 & \cdots & 0 & C_{i}^{T} & 0 & \cdots & 0\\ {}* & * & -\gamma_{i}^{2} & 0 & 0 & \cdots & 0 & 0 & 0 & \cdots & 0\\ {}* & * & * & -\gamma_{i}^{2} & 0 & \cdots & 0 & E_{i}^{T} & 0 & \cdots & 0\\ {}* & * & * & * & -\varepsilon_{m} & \cdots & 0 & 0 & \sqrt{2\varepsilon_{m}} & \cdots & 0\\ {}* & * & * & * & * & \ddots & \vdots & \vdots & \vdots & \ddots & 0\\ {}* & * & * & * & * & * & -\varepsilon_{p} & 0 & 0 & 0 & \sqrt{2\varepsilon_{p}}\\ {}* & * & * & * & * & * & * & -I & 0 & 0 & 0\\ {}* & * & * & * & * & * & * & * & -I & 0 & 0\\ {}* & * & * & * & * & * & * & * & * & -I & 0\\ {}* & * & * & * & * & * & * & * & * & * & -I \end{array}\right]<0$$

*where*$0<\varepsilon_{m},\cdots ,\varepsilon_{p}\ll 1$ ($m,\cdots p\in N_{\bar{i}}$). *Then the error system (6) is asymptotically stable and satisfies* $H_{\infty }$ *performance* ${\left\|r_{i}(k)\right\|}_{2}<\gamma_{i}{\left\|d_{i}(k)\right\|}_{2}$*, and the detection observer gain can be obtained as* $L_{i}=P_{i}^{-1}Y_{i}$.

**Proof** **of** **Theorem** **1.** Consider the following Lyapunov function candidate:

$$V_{i}(k)={e_{i}}^{T}(k)P_{i}e_{i}(k)$$

and the stability of the error system (6) is satisfied if and only if

$$J_{i}=V_{i}(k+1)-V_{i}(k)+{r_{i}}^{T}(k)r_{i}(k)-\gamma_{i}^{2}{d_{i}}^{T}(k)d_{i}(k)<0$$

It yields

$$J_{i}<\begin{array}{c} {e_{i}}^{T}(k)({{\bar{A}}_{i}}^{T}P_{i}{\bar{A}}_{i}-P_{i}+{C_{i}}^{T}C_{i})e_{i}(k)+2{e_{i}}^{T}(k)({{\bar{A}}_{i}}^{T}P_{i}B_{di}+C_{i}^{T}D_{i})d_{i}(k)\\ +2\sum_{j\in N_{\bar{i}}}{e_{i}}^{T}(k){{\bar{A}}_{i}}^{T}P_{i}A_{ij}e_{j}(k)+{d_{i}}^{T}(k)({B_{di}}^{T}P_{i}B_{di}+D_{i}^{T}D_{i}-\gamma_{i}^{2})d_{i}(k)\\ +\sum_{j\in N_{\bar{i}}}{e_{j}}^{T}(k)({A_{ij}}^{T}P_{i}A_{ij}+\varepsilon_{j})e_{j}(k)+2\sum_{j\in N_{\bar{i}}}{e_{j}}^{T}(k){A_{ij}}^{T}P_{i}B_{di}d_{i}(k) \end{array}$$

or equivalently

$${\xi_{i}}^{T}(k)\Xi_{i}\xi_{i}(k)<0$$

where ${\bar{A}}_{i}=A_{ii}-L_{i}C_{i}$ and $\xi (k)={\left[\begin{array}{ccccc} {e_{i}}^{T}(k) & {d_{i}}^{T}(k) & {e_{m}}^{T}(k) & \cdots & {e_{p}}^{T}(k) \end{array}\right]}^{T}$, and

$$\Xi_{i}=\left[\begin{array}{ccccc} {{\bar{A}}_{i}}^{T}P_{i}{\bar{A}}_{i}-P_{i}+C_{i}^{T}C_{i} & A_{ii}^{T}P_{i}B_{di}+C_{i}^{T}D_{i} & {{\bar{A}}_{ii}}^{T}P_{i}A_{im} & \cdots & {{\bar{A}}_{ii}}^{T}P_{i}A_{ip}\\ {}* & {B_{di}}^{T}P_{i}B_{di}+{D_{i}}^{T}D_{i}-\gamma_{i}^{2} & {B_{di}}^{T}P_{i}A_{im} & \cdots & {B_{di}}^{T}P_{i}A_{ip}\\ {}* & * & A_{im}^{T}P_{i}A_{im}+\varepsilon_{m} & \cdots & A_{im}^{T}P_{i}A_{iP}\\ {}* & * & * & \ddots & \vdots \\ {}* & * & * & \cdots & A_{ip}^{T}P_{i}A_{ip}+\varepsilon_{p} \end{array}\right]$$

With applying the Schur complement twice, $\Xi_{i}<0$ can be further expressed as:

$$\left[\begin{array}{ccccccc} -P_{i} & P_{i}{\bar{A}}_{i} & P_{i}B_{di} & P_{i}A_{im} & \cdots & P_{i}A_{ip} & 0\\ {}* & -P_{i} & 0 & 0 & \cdots & 0 & C_{i}^{T}\\ {}* & * & -\gamma_{i}^{2} & 0 & \cdots & 0 & D_{i}^{T}\\ {}* & * & * & \varepsilon_{m} & \cdots & 0 & 0\\ {}* & * & * & * & \ddots & \vdots & \vdots \\ {}* & * & * & * & * & \varepsilon_{p} & 0\\ {}* & * & * & * & * & * & -I \end{array}\right]<0$$

based on this, $\varepsilon_{m}$, ⋯, $\varepsilon_{p}$ are further managed with the use of Schur complement p−1 times. Hence, Theorem 1 can be proven. □

The root mean square (RMS) norm is selected as the residual evaluation function with

$$J_{i,RMS}={\left\|r_{i}(k)\right\|}_{RMS}$$

The threshold, the maximum influence of disturbance on the residual evaluation function without faults, can be computed by

$$J_{i,th}=\underset{\mathrm{fault}-\mathrm{free}}{\sup}{\left\|r_{i}(k)\right\|}_{RMS}$$

Then, the fault detection decision logic can be described by

$$\left\{ \begin{array}{l} J_{i,RMS}>J_{i,th}\Rightarrow \varepsilon_{i}=1\\ J_{i,RMS}\leq J_{i,th}\Rightarrow \varepsilon_{i}=0 \end{array}\right.$$

where $\varepsilon_{i}=1$ denotes that the *i*th subsystem will generate an alarm signal which will be broadcast by the cloud processing unit, and $\varepsilon_{i}=0$ denotes that the *i*th subsystem is healthy.

### 3.2. The Design of Fault Isolation Observer

According to the above fault detection results, the alarming subsystems are firstly put into the fault set and then fault isolation is only conducted in the fault set. Meanwhile, the FIO given in Equation (4) takes advantage of the output signals from sensors as well as the input signals to generate state estimation ${{\hat{x}}_{i}}^{q}(k)$ and fault estimation ${\hat{f}}_{i}(k)$. Based on this, a novel framework of fault isolation is proposed to locate the fault accurately. For this purpose, some error expressions are defined as follows:

$${e_{i}}^{q}(k){=x}_{i}(k)-{{\hat{x}}_{i}}^{q}(k),\;e_{f_{i}}(k)=f_{i}(k)-{\hat{f}}_{i}(k).$$

Then, from Equations (2) and (4), the error dynamical system is described by

(8) $$\left\{ \begin{array}{l} {e_{i}}^{q}(k+1)=(A_{ii}-G_{i}C_{i}){e_{i}}^{q}(k)+\sum_{j\in Alarm}A_{ij}{e_{j}}^{q}(k)+\sum_{\bar{j}\in \bar{Alarm}}A_{i\bar{j}}e_{\bar{j}}(k)+B_{\mathrm{fi}}{e_{f}}_{i}(k)\\ \;\;\;\;+B_{v_{i}}v_{i}(k)-G_{i}E_{i}w_{i}(k)\\ e_{f_{i}}(k+1)=-\Gamma_{i}C_{i}e_{i}^{q}(k)+e_{f_{i}}(k)+\Delta f_{i}(k)-\Gamma_{i}E_{i}w_{i}(k) \end{array}\right.$$

where $\Delta f_{i}(k)=f_{i}(k+1)-f_{i}(k)$ denotes the variation in the fault.

According to the error system (8), the augmented system is as follows:

(9) $$\left\{ \begin{array}{l} {\tilde{e}}_{i}(k+1)={\tilde{A}}_{i}{\tilde{e}}_{i}(k)+{\tilde{B}}_{d_{i}}{\tilde{d}}_{i}(k)\\ e_{f_{i}}(k)={\tilde{E}}_{i}{\tilde{e}}_{i}(k) \end{array}\right.$$

where ${\tilde{e}}_{i}(k)={\left[\begin{array}{cccccc} {e_{i}^{q}}^{T}(k) & e_{f_{i}}^{T}(k) & e_{i}^{T}(k) & e_{m}^{T}(k) & \cdots & e_{p}^{T}(k) \end{array}\right]}^{T}$, ${\tilde{d}}_{i}(k)={\left[\begin{array}{ccc} {\tilde{w}}_{i}^{T}(k) & {\tilde{v}}_{i}^{T}(k) & \Delta {\tilde{f}}_{i}^{T}(k) \end{array}\right]}^{T}$, and ${\tilde{w}}_{i}(k)={\left[\begin{array}{cccc} w_{i}(k) & w_{m}(k) & \;\cdots & w_{p}(k) \end{array}\right]}^{T}$, and ${\tilde{v}}_{i}(k)={\left[\begin{array}{cccc} v_{i}(k) & v_{m}(k) & \;\cdots & v_{p}(k) \end{array}\right]}^{T}$, and $\Delta {\tilde{f}}_{i}(k)={\left[\begin{array}{cccc} \Delta f_{i}(k) & \Delta f_{m}(k) & \;\cdots & \Delta f_{p}(k) \end{array}\right]}^{T}$, and ${\tilde{E}}_{i}=\left[\begin{array}{cccccc} 0 & 1 & 0 & 0 & \cdots & 0 \end{array}\right]$, and ${\tilde{A}}_{i}=\left[\begin{array}{cccccc} A_{ii}-G_{i}C_{i} & B_{f_{i}} & 0 & A_{im} & \cdots & A_{ip}\\ -\Gamma_{i}C_{i} & 1 & 0 & 0 & \cdots & 0\\ 0 & 0 & A_{ii}-L_{i}C_{i} & A_{im} & \cdots & A_{ip}\\ 0 & 0 & A_{mi} & A_{mm}-L_{m}C_{m} & \cdots & A_{mp}\\ \vdots & \vdots & \vdots & \vdots & \ddots & \vdots \\ 0 & 0 & A_{pi} & A_{pm} & \cdots & A_{pp}-L_{p}C_{p} \end{array}\right]$, and ${\tilde{B}}_{d_{i}}=\left[\begin{array}{cccccccccccc} -G_{i}E_{i} & 0 & \cdots & 0 & B_{v_{i}} & 0 & \cdots & 0 & 0 & 0 & \cdots & 0\\ -\Gamma_{i}E_{i} & 0 & \cdots & 0 & 0 & 0 & \cdots & 0 & 1 & 0 & \cdots & 0\\ -L_{i}E_{i} & 0 & \cdots & 0 & B_{v_{i}} & 0 & \cdots & 0 & 0 & 0 & \cdots & 0\\ 0 & -L_{m}E_{m} & \cdots & 0 & 0 & B_{v_{m}} & \cdots & 0 & 0 & 0 & \cdots & 0\\ \vdots & \vdots & \vdots & \vdots & \vdots & \vdots & \vdots & \vdots & \vdots & \vdots & \ddots & \vdots \\ 0 & 0 & \cdots & -L_{p}E_{p} & 0 & 0 & 0 & B_{v_{p}} & 0 & 0 & \cdots & 0 \end{array}\right]$. Thus, the asymptotic stability and $H_{\infty }$ performance of the error system given in Equation (9) are ensured in the following theorem.

**Theorem** **2.** *For a given scalar* $\delta_{i}>0$ *, if there exist symmetric matrices* ${\tilde{P}}_{ii}={\tilde{P}}_{ii}^{T}>0$ *,* ${\tilde{Q}}_{ii}={\tilde{Q}}_{ii}^{T}>0$ *such that the following condition holds*

(10) $$\left[\begin{array}{cccc} -{\tilde{P}}_{i} & \psi_{i} & \Theta_{i} & 0\\ {}* & -{\tilde{P}}_{i} & 0 & {\tilde{E}}_{i}\\ {}* & * & -\delta_{i} & 0\\ {}* & * & * & -\delta_{i} \end{array}\right]<0$$

where ${\tilde{P}}_{i}=diag\left\{\begin{array}{cccccc} {\tilde{P}}_{ii} & {\tilde{Q}}_{ii} & I & I & \cdots & I \end{array}\right\}$, $\psi_{i}=\left[\begin{array}{cccccc} {\tilde{P}}_{ii}A_{ii}-{\tilde{X}}_{i}C_{i} & {\tilde{P}}_{ii}B_{f_{i}} & 0 & {\tilde{P}}_{ii}A_{im} & \cdots & {\tilde{P}}_{ii}A_{ip}\\ -{\tilde{Y}}_{i}C_{i} & {\tilde{Q}}_{ii} & 0 & 0 & \cdots & 0\\ 0 & 0 & A_{ii}-L_{i}C_{i} & A_{im} & \cdots & A_{ip}\\ 0 & 0 & A_{mi} & A_{mm}-L_{m}C_{m} & \cdots & A_{mp}\\ \vdots & \vdots & \vdots & \vdots & \ddots & \vdots \\ 0 & 0 & A_{pi} & A_{pm} & \cdots & A_{pp}-L_{p}C_{P} \end{array}\right]$*,* $\Theta_{i}=\left[\begin{array}{cccccccccccc} -{\tilde{X}}_{i}E_{i} & 0 & \cdots & 0 & {\tilde{P}}_{ii}B_{v_{i}} & 0 & \cdots & 0 & 0 & 0 & \cdots & 0\\ -{\tilde{Y}}_{i}E_{i} & 0 & \cdots & 0 & 0 & 0 & \cdots & 0 & {\tilde{Q}}_{ii} & 0 & \cdots & 0\\ -L_{i}E_{i} & 0 & \cdots & 0 & B_{v_{i}} & 0 & \cdots & 0 & 0 & 0 & \cdots & 0\\ 0 & -L_{m}E_{m} & \cdots & 0 & 0 & B_{v_{m}} & \cdots & 0 & 0 & 0 & \cdots & 0\\ \vdots & \vdots & \ddots & \vdots & \vdots & \vdots & \ddots & \vdots & \vdots & \vdots & \ddots & \vdots \\ 0 & 0 & \cdots & -L_{p}E_{p} & 0 & 0 & \cdots & B_{v_{p}} & 0 & 0 & \cdots & 0 \end{array}\right]$*, then the error dynamics (9) satisfy the* $H_{\infty }$ *performance index* ${\left\|e_{f_{i}}(k)\right\|}_{2}<\delta_{i}{\left\|{\tilde{d}}_{i}(k)\right\|}_{2}$. *Further, the weighting matrix and isolation observer gain are given as* $\Gamma_{i}={\tilde{Q}}_{ii}^{-1}{\tilde{Y}}_{i}$ *and* $G_{i}={\tilde{P}}_{ii}^{-1}{\tilde{X}}_{i}$ *respectively.*

**Proof** **of** **Theorem** **2.** Consider the following Lyapunov function candidate:

$${\tilde{V}}_{i}(k)={\tilde{e}}_{i}^{T}(k){\tilde{P}}_{i}{\tilde{e}}_{i}(k)\;$$

and the stability of the error system Equation (9) is satisfied if and only if

$${\tilde{J}}_{i}={\tilde{V}}_{i}(k+1)-{\tilde{V}}_{i}(k)+e_{f_{i}}^{T}(k)e_{f_{i}}(k)-\delta_{i}^{2}{\tilde{d}}_{i}^{T}(k){\tilde{d}}_{i}(k)<0\;$$

It yields

$$\begin{array}{l} {\tilde{e}}_{i}^{T}(k)({\tilde{A}}_{i}^{T}{\tilde{P}}_{i}{\tilde{A}}_{i}+{\tilde{E}}_{i}^{T}{\tilde{E}}_{i}-{\tilde{P}}_{i}){\tilde{e}}_{i}(k)+{\tilde{e}}_{i}^{T}(k){\tilde{A}}_{i}^{T}{\tilde{P}}_{i}{\tilde{B}}_{d_{i}}{\tilde{d}}_{i}(k)\\ +{\tilde{d}}_{i}^{T}(k){\tilde{B}}_{d_{i}}^{T}{\tilde{P}}_{i}{\tilde{A}}_{i}{\tilde{e}}_{i}(k)+{\tilde{d}}_{i}^{T}(k)({\tilde{B}}_{d_{i}}^{T}{\tilde{P}}_{i}{\tilde{B}}_{d_{i}}-\delta_{i}^{2}){\tilde{d}}_{i}(k) \end{array}<0\;$$

or equivalently

(11) $$\left[\begin{array}{cccc} -{\tilde{P}}_{i} & {\tilde{P}}_{i}{\tilde{A}}_{i} & {\tilde{P}}_{i}{\tilde{B}}_{d_{i}} & 0\\ {}* & -{\tilde{P}}_{i} & 0 & {\tilde{E}}_{i}^{T}\\ {}* & * & -\delta_{i} & 0\\ {}* & * & * & -\delta_{i} \end{array}\right]<0$$

Theorem 2 can be proven by substituting ${\tilde{P}}_{i}$, ${\tilde{A}}_{i}$ and ${\tilde{B}}_{d_{i}}$ into Equation (11). □

Furthermore, the residual evaluation in the fault set is carried out again. The RMS norm is chosen as the residual evaluation function with

$$J_{i,RMS}^{q}={\left\|{r_{i}}^{q}(k)\right\|}_{RMS}$$

and the threshold, similar to previous subsections, is calculated as

$$J_{i,th}^{q}=\underset{\mathrm{fault}-\mathrm{free}}{\sup}{\left\|r_{i}^{q}(k)\right\|}_{RMS}$$

Different from the conventional scheme of decision logic, fault isolation decision logic in this subsection is described by

$$\left\{ \begin{array}{l} J_{i,RMS}^{q}\leq J_{i,th}^{q}\Rightarrow faulty\\ J_{i,RMS}^{q}>J_{i,th}^{q}\Rightarrow fault-free \end{array}\right.$$

and fault compensation is responsible for this.

**Remark** **5.** *Alarm signals from FDOs are broadcasted by the cloud processing unit. In order to realize fault isolation, the alarming subsystems then further employ FIOs in turn after the use of FDOs. Meanwhile, the healthy subsystems, not generating alarm signals, use FDOs at all times.*

### 3.3. The Design of Cooperative Fault-Tolerant Controller

In the two previous subsections, fault detection and isolation have been realized by the design of the fault detection and isolation observer. However, the stability of the whole system cannot be guaranteed because the fault is unsolved during fault diagnosis. For this reason, a cooperative fault-tolerant control scheme for the distributed interconnected system is presented in this subsection.

*To ensure that a distributed interconnected system with an actuator failure can maintain stability, the optimal cooperative fault-tolerant control law is designed as* $u_{i}(k)=-R_{i}^{-1}B_{ii}^{T}K_{i}{\hat{x}}_{zi}(k)-R_{i}^{-1}B_{ii}^{T}S_{i}(k)$ *, where the local control gain and cooperative compensation vector are determined from*

(12) $$K_{i}=Q_{i}+{A_{ii}}^{T}K_{i}A_{ii}-{A_{ii}}^{T}K_{i}B_{ii}{\left(R_{i}+{B_{ii}}^{T}K_{i}B_{ii}\right)}^{-1}{B_{ii}}^{T}K_{i}A_{ii}$$

(13) $$S_{i}(k+1)={\left[A_{ii}^{T}-A_{ii}^{T}K_{i}B_{ii}{\left(R_{i}+B_{ii}^{T}K_{i}B_{ii}\right)}^{-1}B_{ii}^{T}\right]}^{-1}S_{i}(k)-K_{i}\sum_{j\in N_{\bar{i}}}\left(A_{ij}x_{j}(k)+B_{ij}u_{j}(k)\right)$$

**Proof** **of** **Theorem** **3.** The global optimization problem for the control of the distributed interconnected system with a failure is a quadratic function related to the state and input vectors, which can be given as

(14) $$\min\underset{u_{i}}{M}=\sum_{i=1}^{N}\frac{1}{2}(x_{i}^{T}(k)Q_{i}x_{i}(k)+u_{i}^{T}(k)R_{i}u_{i}(k))$$

where $Q_{i}$ is a positive semidefinite matrix.

When one subsystem fails, the effect of cooperative compensation can be achieved by the Hamiltonian for each subsystem

(15) $$\begin{array}{l} H_{i}=\frac{1}{2}({x_{i}}^{T}(k)Q_{i}x_{i}(k)+{u_{i}}^{T}(k)R_{i}u_{i}(k))+{\lambda_{i}}^{T}(k+1)(A_{ii}x_{i}(k)+B_{ii}u_{i}(k)\\ \;\;+\sum_{j\in N_{\bar{i}}}A_{ij}x_{j}(k)+\sum_{j\in N_{\bar{i}}}B_{ij}u_{j}(k)) \end{array}$$

where $\lambda_{i}(k+1)$ is the adjoint vector.

According to Equation (15), the necessary optimality conditions, obtained from the optimal control theory [28], are

(16) $$\frac{\partial H_{i}}{\partial x_{i}}=Q_{i}x_{i}(k)+{A_{ii}}^{T}\lambda_{i}(k+1)=\lambda_{i}(k)$$

(17) $$\frac{\partial H_{i}}{\partial u_{i}}=R_{i}u_{i}(k)+{B_{ii}}^{T}\lambda_{i}(k+1)=0$$

Further, the cooperative compensation is calculated through a feedback control, which can be described by

(18) $$\lambda_{i}(k)=K_{i}{\hat{x}}_{i}(k)+S_{i}(k)$$

Substituting Equations (18) and (2) without the fault $f_{i}(k)$ and the noise $v_{i}(k)$ into Equation (16) yields

(19) $$\begin{array}{l} \lambda_{i}(k)=(Q_{i}+{A_{ii}}^{T}K_{i}A_{ii})x_{i}(k)+{A_{ii}}^{T}K_{i}B_{ii}u_{i}(k)+\sum_{j\in N_{\bar{i}}}{A_{ii}}^{T}K_{i}A_{ij}x_{j}(k)\\ \;\;\;+\sum_{j\in N_{\bar{i}}}{A_{ii}}^{T}K_{i}B_{ij}u_{j}(k)+{A_{ii}}^{T}S_{i}(k+1) \end{array}$$

By combining Equations (18) and (2) without the fault $f_{i}(k)$ and the noise $v_{i}(k)$, Equation (17) can be re-written as

(20) $$\begin{array}{l} u_{i}(k)=-{\left(R_{i}+{B_{ii}}^{T}K_{i}B_{ii}\right)}^{-1}{B_{ii}}^{T}K_{i}A_{ii}x_{i}(k)-\sum_{j\in N_{\bar{i}}}{\left(R_{i}+{B_{ii}}^{T}K_{i}B_{ii}\right)}^{-1}{B_{ii}}^{T}K_{i}A_{ij}x_{j}(k)\\ \;\;-\sum_{j\in N_{\bar{i}}}{\left(R_{i}+{B_{ii}}^{T}K_{i}B_{ii}\right)}^{-1}{B_{ii}}^{T}K_{i}B_{ij}u_{j}(k)-{\left(R_{i}+{B_{ii}}^{T}K_{i}B_{ii}\right)}^{-1}{B_{ii}}^{T}S_{i}(k+1) \end{array}$$

Moreover, substituting Equation (20) into Equation (19) leads to

(21) $$\begin{array}{c} \lambda_{i}(k)=[Q_{i}+{A_{ii}}^{T}K_{i}A_{ii}-{A_{ii}}^{T}K_{i}B_{ii}{\left(R_{i}+{B_{ii}}^{T}K_{i}B_{ii}\right)}^{-1}{B_{ii}}^{T}K_{i}A_{ii}]x_{i}(k)\\ +[\sum_{j\in N_{\bar{i}}}{A_{ii}}^{T}K_{i}A_{ij}-{A_{ii}}^{T}K_{i}B_{ii}{\left(R_{i}+{B_{ii}}^{T}K_{i}B_{ii}\right)}^{-1}{B_{ii}}^{T}K_{i}A_{ij}]x_{j}(k)\\ +[\sum_{j\in N_{\bar{i}}}{A_{ii}}^{T}K_{i}B_{ij}-{A_{ii}}^{T}K_{i}B_{ii}{\left(R_{i}+{B_{ii}}^{T}K_{i}B_{ii}\right)}^{-1}{B_{ii}}^{T}K_{i}B_{ij}]u_{j}(k)\\ +[{A_{ii}}^{T}-{A_{ii}}^{T}K_{i}B_{ii}{\left(R_{i}+{B_{ii}}^{T}K_{i}B_{ii}\right)}^{-1}{B_{ii}}^{T}]S_{i}(k+1) \end{array}$$

Theorem 3 can be proven by comparing Equations (18) and (21). □

## 4. Simulation Example

**Figure 4:**
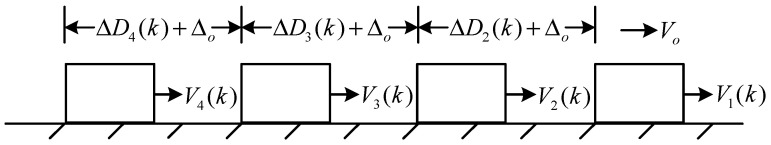
Intelligent unmanned vehicle platooning made up of four vehicles.

In this section, the proposed fault diagnosis and cooperative fault-tolerant control scheme is applied to the simplified model of intelligent unmanned vehicle platooning [29], which is shown in Figure 4. A desired separation distance $\Delta_{o}$ between adjacent vehicles, and a desired average velocity $V_{o}$ should be assigned under normal operating conditions. Furthermore, the variable $\Delta D_{i}(k)$ (i=2,3,4) represents the deviation from the desired separation distance while the variable $\Delta V_{i}(k)$ (i=1,2,3,4) represents the deviation from the desired velocity. $\Delta d_{i}(k)$ is the real separation distance between the *i*th and i−1th vehicle at time k, and $V_{i}(k)$ is the real velocity of the *i*th vehicle at time k. Therefore, the state vector and output vector in Equation (1) are $x(k)={\left[\begin{array}{ccccccc} \Delta V_{4}(k) & \Delta D_{4}(k) & \Delta V_{3}(k) & \Delta D_{3}(k) & \Delta V_{2}(k) & \Delta D_{2}(k) & \Delta V_{1}(k) \end{array}\right]}^{T}$ and $y(k)=[\begin{array}{cc} \Delta D_{4}(k) & \Delta D_{3}(k) \end{array}$ ${\begin{array}{cc} \Delta D_{2}(k) & \Delta V_{1}(k) \end{array}]}^{T}$ respectively, where $\Delta D_{i}(k)=$$\Delta d_{i}(k)-\Delta_{o}$ and $\Delta V_{i}(k)=V_{i}(k)-V_{o}$.

The motion of each vehicle is characterized firstly by differential equations with the help of Newton’s second law, and then the state-space representation of the four vehicles platooning can be acquired through expanding the nonlinear term in a Taylor series expansion. The system matrices of the four vehicles platooning are given as follows:

$$A=\left[\begin{array}{ccccccc} 0.9048 & 0 & 0 & 0 & 0 & 0 & 0\\ 0.0952 & 1 & -0.0952 & 0 & 0 & 0 & 0\\ 0 & 0 & 0.9048 & 0 & 0 & 0 & 0\\ 0 & 0 & 0.0952 & 1 & -0.0952 & 0 & 0\\ 0 & 0 & 0 & 0 & 0.9048 & 0 & 0\\ 0 & 0 & 0 & 0 & 0.0952 & 1 & -0.0952\\ 0 & 0 & 0 & 0 & 0 & 0 & 0.9048 \end{array}\right]\;B=\left[\begin{array}{cccc} 0.0952 & 0 & 0 & 0\\ 0.0048 & -0.0048 & 0 & 0\\ 0 & 0.0952 & 0 & 0\\ 0 & 0.0048 & -0.0048 & 0\\ 0 & 0 & 0.0952 & 0\\ 0 & 0 & 0.0048 & -0.0048\\ 0 & 0 & 0 & 0.0952 \end{array}\right]’\;B_{v}=0.1B_{f},\;C=\left[\begin{array}{ccccccc} 0 & 1 & 0 & 0 & 0 & 0 & 0\\ 0 & 0 & 0 & 1 & 0 & 0 & 0\\ 0 & 0 & 0 & 0 & 0 & 1 & 0\\ 0 & 0 & 0 & 0 & 0 & 0 & 1 \end{array}\right],\;E=\left[\begin{array}{cccc} 1 & 0 & 0 & 0\\ 0 & 1 & 0 & 0\\ 0 & 0 & 1 & 0\\ 0 & 0 & 0 & 1 \end{array}\right].\;$$

According to Theorem 1, the four parameters $\gamma_{1}$, $\gamma_{2}$, $\gamma_{3}$, and $\gamma_{4}$ are computed as 1.1339, 1.1339, 1.1339, and 1.0003 respectively, and the detection observer gains are as follows:

$$L_{1}=\left[\begin{array}{c} 1.1439\times 10^{-4}\\ 0.2222 \end{array}\right],\;L_{2}=\left[\begin{array}{c} 1.1439\times 10^{-4}\\ 0.2222 \end{array}\right],\;L_{3}=\left[\begin{array}{c} 1.1439\times 10^{-4}\\ 0.2222 \end{array}\right],\;L_{4}=4.5233\times 10^{-4}$$

Then, by solving the condition in Theorem 2, we can obtain the $H_{\infty }$ performance levels $\delta_{4}=1.8338$, $\delta_{3}=1.8633$, and the isolation observer parameters

$$G_{4}=0.9048,\;G_{3}={\left[\begin{array}{cc} 0.0044 & 0.6059 \end{array}\right]}^{T},\;\Gamma_{4}=1,\;\Gamma_{3}=8.652\times 10^{-6}.\;$$

Further, by solving Riccati Equation (9), the local optimal gains can be obtained as:

$$K_{1}=\left[\begin{array}{cc} 0.3112 & 0.3113 \end{array}\right],\;K_{2}=\left[\begin{array}{cc} 0.3112 & 0.3113 \end{array}\right],\;K_{3}=\left[\begin{array}{cc} 0.3112 & 0.3113 \end{array}\right],\;K_{4}=0.0463.\;$$

In the simulation, the process and measurement noise are assumed as $v(k)=w(k)={\left[\begin{array}{cccc} 0.1 & 0.1 & 0.1 & 0.1 \end{array}\right]}^{T}\cdot U\left[\begin{array}{cc} -1 & 1 \end{array}\right]$. Meanwhile, a fault has occurred in the 1st vehicle and is chosen as

$$f_{1}(k)=\left\{ \begin{array}{l} \begin{array}{cc} 0, & k<30s \end{array}\\ \begin{array}{cc} 0.8, & k\geq 30s \end{array} \end{array}\right.$$

**Figure 5:**
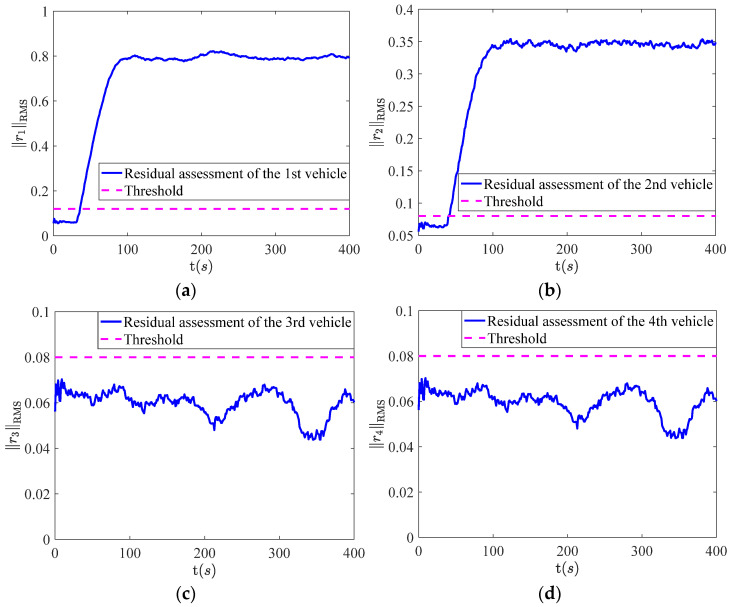
The results of fault detection, where (**a**) represents the 1st vehicle; (**b**) represents the 2nd vehicle; (**c**) represents the 3rd vehicle; (**d**) represents the 4th vehicle.

The simulation results of fault detection are depicted in Figure 5a–d. It can be observed that the 1st and 2nd vehicles generate alarm signals and the 3rd and 4th vehicles do not generate alarm signals. Thus, the fault detection logic table can be listed as follows:

The simulation results of fault detection are depicted in Figure 5a–d. It can be observed that the 1st and 2nd vehicles generate alarm signals and the 3rd and 4th vehicles don’t generate alarm signals. So the fault detection logic table can be listed as Table 1.

It indicates that the faulty vehicle is located in alarming vehicles and the 3rd and 4th vehicles are healthy because of no alarm signals. Hence, the fault set is defined as {the 1st vehicle, the 2nd vehicle}. The next step is to determine whether the faulty vehicle is the 1st vehicle or the 2nd vehicle. For this purpose, fault isolation for the 1st vehicle and the 2nd vehicle are carried out in turn.

**Figure 6:**
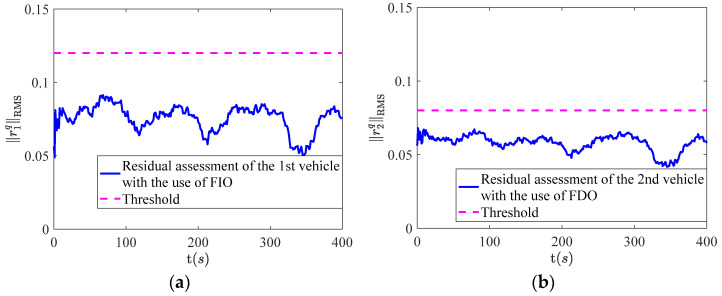
The results of fault isolation, where (**a**) represents the 1st vehicle with the use of FIO; (**b**) represents the 2nd vehicle with the use of FDO.

**Figure 7:**
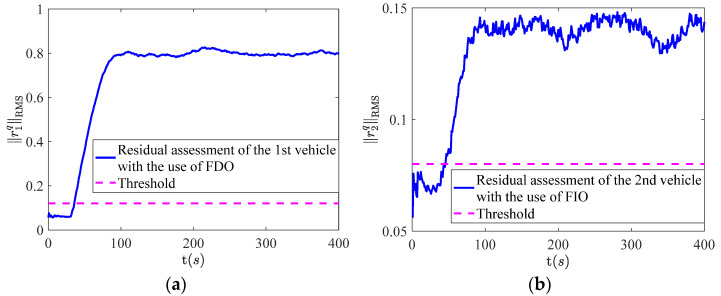
The results of fault isolation, where (**a**) represents the 1st vehicle with the use of FDO; (**b**) represents the 2nd vehicle with the use of FIO.

The simulation results of fault isolation for the 1st vehicle and 2nd vehicle are shown in Figure 6 and Figure 7 respectively. It can be seen from Figure 6a,b that the residual assessment values of the 1st and 2nd vehicle are both less than the threshold. However, the residual assessment values of the 1st and 2nd vehicle are both over the threshold in Figure 7a,b after the occurrence of the fault. Based on this, the fault isolation logic table can be listed as Table 2.

Combing the simulation results and the fault isolation logic, it can be found that the faulty vehicle is the 1st vehicle.

**Figure 8:**
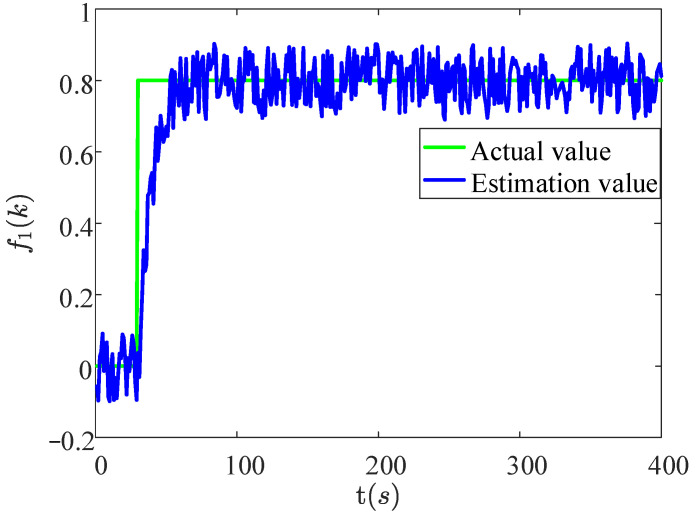
The actual value and estimation value of $f_{1}(k)$.

Meanwhile, it also can be found from Figure 8 that the fault estimation value of the 1st vehicle can follow the fault value rapidly and accurately in a short time.

**Figure 9:**
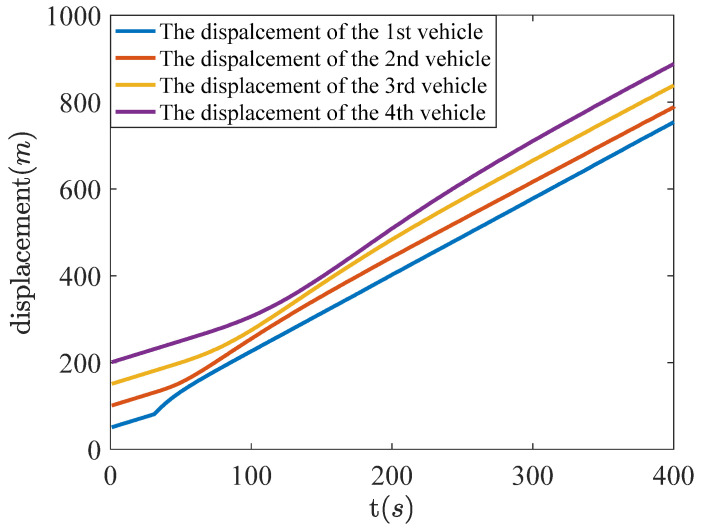
Displacements of four vehicles with the use of fault-tolerant control scheme.

In order to guarantee the stability of the whole intelligent unmanned vehicle platooning, a cooperative controller with fault-tolerant ability is applied to the 1st vehicle and LQR controllers are used for the other three vehicles, and fault-tolerant results are further shown in Figure 9. It is obvious that the malfunction of the 1st vehicle brings about fault propagation among intelligent unmanned vehicle platooning, so that the displacement curves of the other three vehicles are no longer parallel with each other for a period of time. However, the displacement curves of the four vehicles are parallel again under the action of cooperative fault-tolerant control, which demonstrates the effectiveness of the fault-tolerant control scheme proposed in this paper.

## 5. Conclusions

In this paper, a distributed fault diagnosis and cooperative fault-tolerant control framework was developed. To be specific, a fault detection observer was first designed for each subsystem, and the generated alarm signals were broadcast by the cloud processing unit. After that, fault isolation observers and isolation decision logic were used for alarming subsystems in turn to locate the fault accurately. Furthermore, the control unit with the effect of cooperative compensation was constructed to avoid the system instability caused by the faulty subsystem.

It is notable that the scheme in this paper can be employed if and only if a single fault occurs in the system, so it is challenging to study actuator faults as well as sensor faults simultaneously for a distributed interconnected system. Meanwhile, it is very meaningful to combine our proposed method with the distinguished technologies in [8,18,19,20], focusing on optimizing shared information among subsystems, so as to reduce communication costs. These may represent the directions of our future work.

## Figures and Tables

**Table 1:** Fault detection logic.

Numbers	Decision Results
The 1st vehicle	1
The 2nd vehicle	1
The 3rd vehicle	0
The 4th vehicle	0

**Table 2:** Fault isolation logic.

Types	Numbers	Decision Results
Fault isolation for the 1st vehicle	The 1st vehicle (FIO)	0
	The 2nd vehicle (FDO)	0
Fault isolation for the 2nd vehicle	The 1st vehicle (FDO)	1
	The 2nd vehicle (FIO)	1

## Data Availability

Not applicable.
